# Post-COVID-19 Demyelinating Disease and Its Effect on the Lower Urinary Tract: A Rare Case of a 14-Year-Old Man

**DOI:** 10.7759/cureus.49022

**Published:** 2023-11-18

**Authors:** Ioannis Tsikopoulos, Georgios Antoniadis, Charalampos Konstantinidis, Michalis Samarinas

**Affiliations:** 1 Urology, General University Hospital of Larissa, Larissa, GRC; 2 Urology and Neuro-urology, National Rehabilitation Center, Athens, GRC

**Keywords:** clinical case report, urodynamic, neuro-urology, covid-19, demyelinating neurological disorder

## Abstract

The COVID-19 pandemic caused by severe acute respiratory coronavirus 2 (SARS-CoV-2) has led to a wide range of manifestations, including urological issues. Patients with COVID-19 frequently experience complications, such as acute kidney injury (AKI) and thromboembolism. Neurological problems, including demyelination in the central and peripheral nervous systems, have also been reported in COVID-19 cases. This neurological damage can be attributed to the virus's neurotropic and neuro-invasive properties. This case study presents a 14-year-old patient who developed severe lower urinary tract symptoms following a COVID-19 infection, leading to a demyelinating disease affecting the lower urinary tract. The patient was managed successfully with specialized neuro-urological care, highlighting the importance of multidisciplinary collaboration in managing post-COVID-19 complications. Clinicians need to be vigilant about potential neurological manifestations in COVID-19 patients, including those affecting the urinary system, and patients should seek specialized medical attention for persistent symptoms.

## Introduction

The new coronavirus, known as severe acute respiratory coronavirus 2 (SARS-CoV-2), was the cause of an epidemic outbtreak in December 2019 in Wuhan, China. Since then, there was an extensive investigation and many reports on the virus's effects on multiple systems.

From a urological point of view, there are also manifestations attributed to COVID-19. Acute kidney injury (AKI) is frequently reported in patients with COVID-19 (1-46%). Severe structural changes in the kidneys of patients with COVID-19 can be found in pathological examinations and underlying mechanisms include cytokine storm-induced systemic inflammatory response syndrome (SIRS) and direct cytopathic effects [1]. Thromboembolism is a major complication of COVID-19 and has been reported in 31% of patients in the ICU with COVID-19 pneumonia. Prostate and renal infarction, as well as priapism, were reported as thromboembolic complications [2]. As far as the lower urinary tract, there are increased reported symptoms of frequency, nocturia, and lower-grade International Prostate Symptom Score (IPSS) in patients with COVID-19. The detection of SARS-CoV-2 in urine is rare (5% of patients), but it can persist up to 52 days after the onset of the disease [3]. In 85% of COVID-19 patients, secondary hypogonadism was observed, although the impact of testosterone levels on survival remains a topic of debate. Examination of the testes from COVID-19 patients revealed significant alterations, such as interstitial swelling, localized shrinkage, and the presence of red blood cells. SARS-CoV-2 was identified in testicular tissues through electron microscopy, yet in most studies, it could not be detected in semen. Patients with COVID-19 have been reported to experience reduced sperm count and diminished overall sperm motility [4].

Statistics have shown that over 33% of COVID-19 patients faced neurological issues during the initial stage, and a similar percentage continued to experience these problems six months after being infected [5]. Regarding demyelinating diseases, there have been several reports of multiple demyelination occurrences in both the central nervous system (CNS) and peripheral nervous system (PNS) as a result of the COVID-19 infection. CNS demyelination has long been linked with viral infections, and SARS-CoV-2 infection has been concordant with this association [6].

## Case presentation

A 14-year old male patient presented to the emergency department with bilateral lower extremity weakness and urinary retention for six hours. The patient's notable clinical findings included diminished superficial sensation corresponding to the T6 dermatome level, coupled with a decreased deep sensation in both the lower extremities and symmetrical distal sensory loss (to pinprick). Moreover, the patient exhibited severely reduced muscle tone (flaccidity) and absent muscle stretch tendon reflexes in both the lower extremities and lack of sensation of bladder filling. The patient's Glascow Coma Scale (GCS) and Extended Disability Status Scale (EDSS) were 15/15 and 8, respectively. Both the bulbocavernosus reflex (BCR) and sphincter tone were absent and the patient manifested bilateral extension of the plantar reflex.

**Figure 1:**
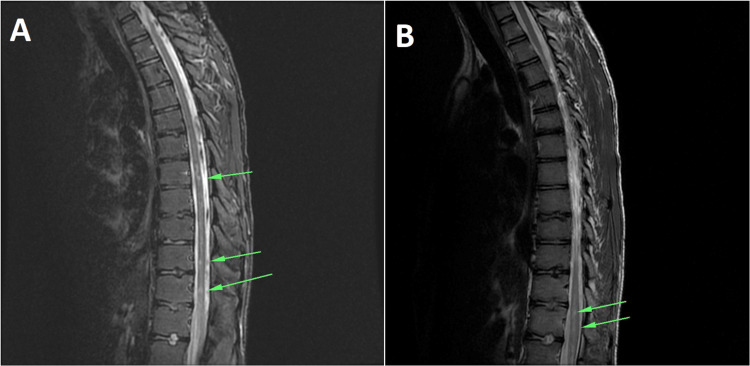
(A) T2-weighted sagittal MRI view of the thoracolumbar spine. (B) T1-weighted sagittal MRI view of the thoracolumbar spine. The arrows are depicting demyelinating lesions, which are located below the T7 vertebra level and and until the L1/L2 spine levels.

**Figure 2:**
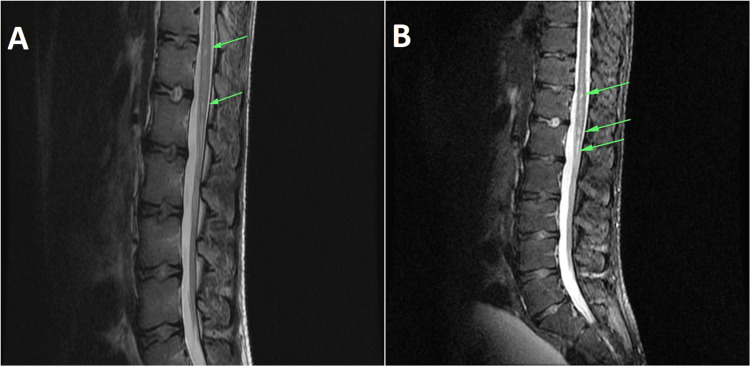
(A) T1-weighted sagittal MRI view of the thoracolumbosacral spine. (B) T2-weighted MRI of the thoracolumbosacral spine. The arrows are showing demyelination.

Regarding medical history, he reported a mildly symptomatic COVID-19 infection one month ago, with a deteriorating back pain, extending to both legs. Pediatricians classified those symptoms as flaccid paraplegia after a viral infection and requested both an MRI and microbiological investigation. Nothing abnormal was detected in the brain, but the spine MRI depicted patchy T2/STIR hyperintense areas of signal abnormality lesions below the thoracic (T7) level (Figures 1A, 1B) and until L1/L2 spine levels (Figures 2A, 2B) indicative of demyelination/myelitis. Cerebrospinal fluid (CSF) analysis was negative for infections (cultures and gram stain) and viruses (PCR for herpes simple virus, varicella zoster virus, measles virus, influenza virus, human cytomegalovirus, enteroviruses, and mumps virus), while immunological tests were unremarkable. In addition, blood cultures were collected and were negative for bacteria and fungi. The patient was subsequently submitted to the pediatric department bearing a Foley catheter and was commenced on IV corticosteroids. A regimen of physiotherapeutic sessions was implemented with the objective of enhancing strength in both the upper and lower limbs and re-establishing ambulatory skills.

**Figure 3:**
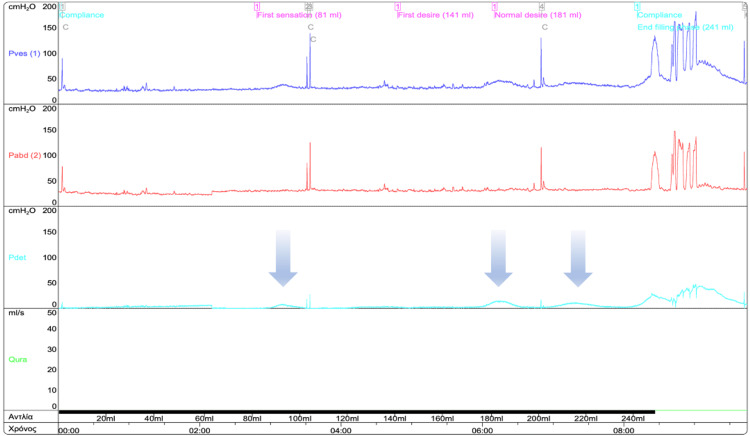
Pressure-flow study Urodynamic study showing a phasic overactive detrusor muscle of the bladder during the filling phase of the examination (arrows) and abdominal strain, low detrusor pressure, and failure to achieve bladder emptying during the voiding phase.

While inpatient, a neuro-urological evaluation request was made, and the patient was referred to our urodynamics unit for investigation and consultation. A pressure flow study was conducted and revealed phasic overactive detrusor muscle of the bladder during the filling phase of the examination and abdominal strain, low detrusor pressure, and failure to achieve bladder emptying during the voiding phase (Figure 3). Furthermore, low detrusor contractility was recorded along with straining and detrusor sphincter dyssynergia. We hypothesized that the cause of those results was a secondary damage in terms of the neurological post-COVID-19 lesion. Cystoscopy was unremarkable, and during a voiding cystourethrogram (VCUG), no vesicoureteral reflux (VUR) was depicted.

The patient was advised to initiate treatment with oxybutynin 5 mg once a day in combination with four to five self-catheterizations per day so as to avoid abdominal strain. The short-term follow-up examination a month later revealed a satisfied patient totally compliant with the suggested therapeutic plan and a complication-free treatment.

## Discussion

Mao et al. in a retrospective study observed for the first time 214 COVID-19 patients coming from three COVID-19 centers in China, in an attempt to report characteristic neurologic manifestations of the COVID-19 infection. The cases were sub-grouped into three categories: CNS manifestations (dizziness, headache, impaired consciousness, acute cerebrovascular disease, ataxia, and seizure), PNS manifestations (taste impairment, smell impairment, vision impairment, and nerve pain), and skeletal muscular injury manifestations. This was the first detailed report on the neurologic manifestations of hospitalized patients with COVID-19 [7]. From then on, cases of more complicated and severe neurological conditions have followed. Among them were demyelinating diseases, including more severe, even fulfilling McDonald's criteria for multiple sclerosis (MS) in patients between 20 and 50 years old [8]. Four demyelinating manifestations of either CNS or PNS have been reported in post COVID-19 patients by Ismail and Salama: acute demyelinating encephalomyelitis (ADEM), transverse myelitis (TM), multiple sclerosis-like demyelination, and neuromyelitis optica spectrum disorder (NMOSD) [9].

COVID-19 displays neurotropic and neuro-invasive characteristics, leading to direct neurological harm by binding to angiotensin-converting enzyme-2 (ACE-2) receptors. These receptors are widespread, including in the CNS, allowing the virus to cause damage either through direct interaction or by circulating through Virchow-Robin spaces [10]. In addition, delayed damage to the CNS seems to be triggered by an unintended immune response following the acute infection, ultimately resulting in demyelination in the CNS [11]. An alternate explanation could involve the development of antibodies against myelin prompted by the virus. This para-infectious or post-infectious origin has been observed in numerous cases of Guillain-Barré syndrome following SARS-CoV-2 infection. There is a possibility that SARS-CoV-2 might initiate MS, akin to the established role of the Epstein-Barr virus.

Our patient’s provisional diagnosis was post COVID-19 demyelinating disease (myelitis) as there was a lack of brain inflammation or lesions. We conducted a literature search through the major databases, and to our knowledge, this was the first case of post-COVID-19 demyelinating disease affecting the lower urinary tract in such a dramatic manner. Fortunately, this condition was detected at an early stage and managed properly in a specialized neuro-urology center.

## Conclusions

There are many post-COVID-19 neurologic manifestations that clinicians should be aware of. One of them is the demyelinating disease that seems to possibly affect the lower urinary tract, causing both voiding and storage symptoms and even urge urinary incontinence (UUI) or complete voiding failure like our patient. Post COVID-19 patients should be followed up regularly for their urinary system, including both the lower and upper urinary tracts. Moreover, patients should be well informed of such possibility and therefore seek for specialized medical help in case of persistent urinary tract symptoms. This case also highlights the necessity of multi-specialty collaboration and management and its beneficial effect on the patient’s health.
